# A harmful bite to the heart

**DOI:** 10.1093/ehjcr/ytab513

**Published:** 2022-03-21

**Authors:** Chiara Bernelli, Annamaria Nicolino, Shahram Moshiri

**Affiliations:** Department of Cardiology, Santa Corona Hospital, Via XXV Aprile 38 Pietra Ligure (SV), 17027, Italy

A 60-year-old male was referred to the emergency department after a European snake bite (*Vipera Aspis Francisciredi*) (*Panel* *A*), complaining of vomiting, and mouth’s dryness. The first electocardiogram (ECG) was unremarkable. However, during observation patient developed five episodes of ventricular fibrillation (VT), treated with Direct-Current Defibrillation (DC) shock and Advanced Cardiovascular Life Support (ACLS). Repeated ECG demonstrated anterolateral ST-elevation myocardial infarction (MI) (*Panel B*). In 2018, for an acute coronary syndrome, he underwent everolimus-eluting-stent (EES) implantation on the left anterior descending artery (LAD) and first diagonal (D1) branch with a T-modified-stent-technique, and he was discharged with ticagrelor 180 mg/day for 1 year.

Coronary angiograms revealed a very late EES thrombosis in mid-LAD-D1 (*Panel D*) which was satisfactorily treated with mechanical thrombectomy and kissing balloon inflations with non-compliant balloons (*Panel A*). Intracoronary imaging was not performed to assess the potential mechanisms underlying ST due to refractory VT episodes. The patient was treated with bolus and infusion and integrilin. Ticagrelor 90 mg/b.i.d. followed by 60 mg/b.i.d. after 1 year plus aspirin was planned. He was treated with anti-snake venom (ANTYTOKSYNA JADU ZMIJ) and other supportive measures. The subsequent course was uneventful. The patient developed massive oedema of the limb point, eyelid ptosis associated with weakness of the musculature associated with chewing, with progressive improvement.

**Figure:**
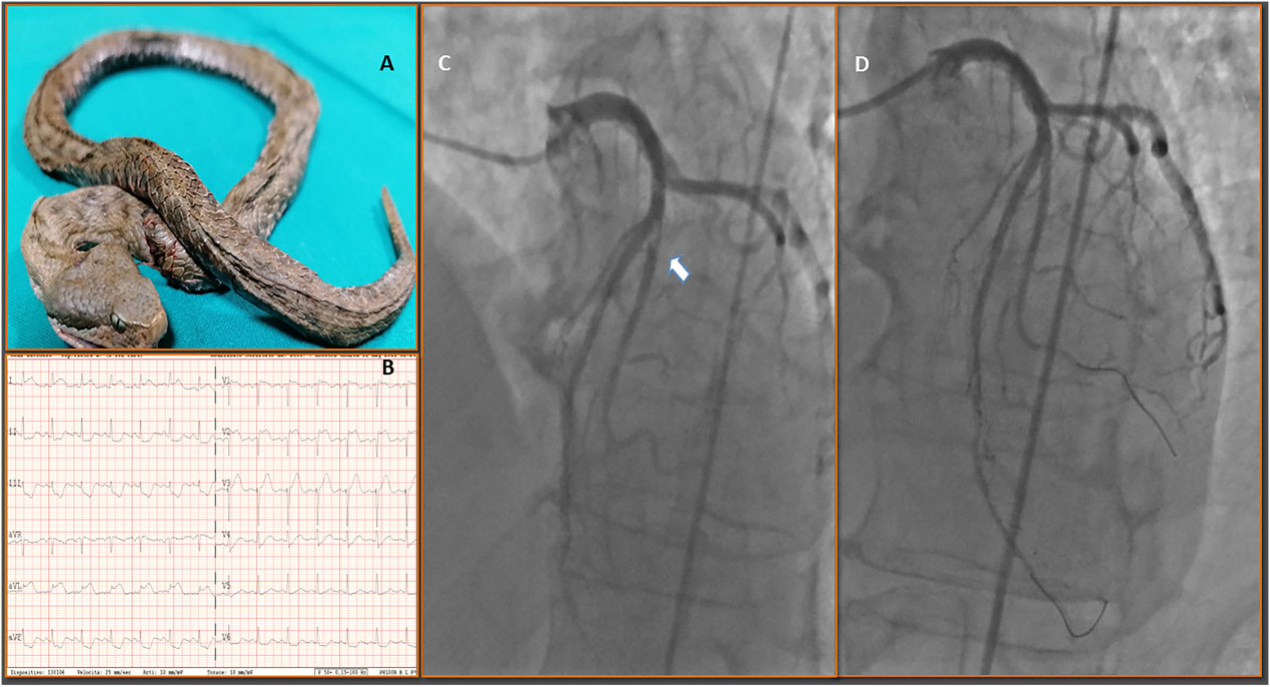


Snakebite can be an unusual cause of acute MI. The direct damages from cytotoxins contained in the snake venom can induce a hypercoagulability state and a hyperadrenergic stimulation with consequent platelet aggregation and coronary vasospasm. Finally, the allergic insult due to bradichinine release after the snake bite could trigger a Kounis type 3 MI.

(*Panel A*) shows a dissected European viper, *Vipera Aspis Francisciredi*, killed by the patient after being bitten; (*Panel B*) repeated ECG after the admission demonstrates ST-elevation anterolateral myocardial infarction (STEMI); (*Panel C*) coronary angiography performed during STEMI illustrates the angiographic appearance of a thrombus (arrow) causing very late stent thrombosis on drug-eluting-stent on the left anterior descending artery (LAD) and first diagonal branch (D1) LAD-D1 bifurcation; (*Panel D*) satisfactory final results after primary percutaneous coronary intervention (PCI) and thrombus aspiration of the infarct-related artery;

